# Ultrasound-Guided Rectus Sheath Block as the Single Anaesthetic Technique for Umbilical Hernia Repair: A Report of 3 Cases

**DOI:** 10.5152/TJAR.2022.1188

**Published:** 2022-04-01

**Authors:** Berna Çalışkan, Çağatay Metin, Öznur Şen

**Affiliations:** 1 Department of Anaesthesiology, İstanbul Haseki Education and Research Hospital, İstanbul, Turkey; 2 Department of Anaesthesiology, İstanbul Sancaktepe Education and Research Hospital, İstanbul, Turkey

**Keywords:** Rectus sheath block, regional anaesthesia, ultrasonography, umbilical hernia repair

## Abstract

In this study, we report 3 cases of ultrasound-guided rectus sheath block used for anaesthetic management of simple periumbilical surgery. We selected 3 patients based on the American Society of Anaesthesiology I-II and defect sizes known to be smaller than 4 cm without peritoneal involvement. We applied a rectus sheath block with 10 mL of 0.5% bupivacaine and 5 mL of 2% lidocaine bilaterally deposited in the space between rectus abdominis and posterior rectus sheath under real-time ultrasonography. Two of our patients tolerated surgery well with minimal sedoanalgesia; however, one of our patients needed dissociative anaesthesia to be compatible because the surgeon found out that the defect was bigger and adjacent to the peritoneum. Rectus sheath block is an underused technique that has the potential to be used as a sole anaesthetic technique in selected cases. So it would be wise to improve and consider rectus sheath block as a valuable tool when there is no better.

**Main Points**

- Rectus sheath block (RSB) can be a valuable alternative to general or spinal anaesthesia as a sole anaesthetic management method for umbilical hernia surgery.
- Selected cases, especially the American Society of Anesthesiology III-IV patients, can benefit from RSB with minimum effect on current physiological status.
- Rectus sheath block provides postoperative effective analgesia and decreases the need for the hospital stay. All of which will result in the effective use of hospital services.

## Introduction

Rectus sheath block (RSB) was first described by Schleich in 1899 to improve analgesia and facilitate surgery involving the anterior abdominal wall from the xiphoid process to symphysis pubis.^1^

Rectus sheath block provides somatic analgesia by blocking the terminal branches of the thoracolumbar nerves within the substance of the rectus abdominis muscle (RAM). In the beginning, it was performed by loss of resistance technique mostly in adults. However, ultrasound has offered a safer guide for peripheric nerve blocks by making layers of the rectus sheath and vascular structures obvious with peritoneal movement below. So; recently, RSB is more commonly used even mostly in pediatric patients.

Rectus sheath block has been used in day-case umbilical hernia surgeries in children as an alternative for the caudal block with less drug used mentioned as protection from systemic toxicity.^2^ Its favor of less motor weakness and urinary retention can also result in early discharge.

Historically, RSB was primarily used as an analgesic adjunct for umbilical hernia repair or laparoscopic gynecologic procedures; however, with the evolving guidance of ultrasound, it is possible to perform more effective blocks by targeting the posterior rectus sheath compartment and visualizing local anaesthetic spread.^3^ Therefore, RSB can be used as a sole anaesthetic agent for a day-case surgery in adults. It would be an invaluable alternative in cases of high-risk patients requiring minor surgeries like umbilical hernias with small defects. 

We herein present 3 cases of open umbilical hernia repair with different defect sizes performed under ultrasound-guided bilateral RSB and intravenous (iv) sedoanalgesia only. 

## Case Presentation

We consulted with the surgical team before surgery to identify the defect size of the umbilical hernias and the extent of the surgery about peritoneal involvement. In this study, 61-year-old male with American Society of Anaesthesiology (ASA) classification I (case 1), 55-year-old ASA II female with stabile concurrent comorbidities of type 2 diabetes mellitus, asthma, and goiter (case 2), and 62-year-old ASA II female with controlled coronary artery disease history (case 3) were evaluated. All 3 patients were approved to have defect sizes smaller than 4 cm without peritoneal involvement and chosen to apply the same ultrasound-guided bilateral RCB. 

Standard monitoring which included electrocardiography, pulse oximetry, and non-invasive blood pressure monitoring was performed in the preoperative room. We applied sedation with 0.03 mg kg-1 midazolam and 1 µg kg-1 fentanyl.

Abdominal skin asepsis was ensured and the ultrasound probe was equipped with a sterile cover to maintain the aseptic block technique. The transducer (high-frequency linear array) is placed just lateral to the umbilicus in a transverse plane. We used a 22-gauge, 80 mm needle positioned inplane to the transducer. Under real-time ultrasonographic guidance, we identified the layers of the anterior abdominal wall focusing particularly on the lateral aspect of linea semilunaris and RAM. We targeted the posterior rectus sheath compartment which is between the posterior border of the RAM but superficial to the posterior rectus sheath. After correcting the area with 1-3 mL of saline, we injected 10 mL of 0.5% bupivacaine and 5 mL of 2% lidocaine. The same procedure is repeated on the contralateral side. All blocks were performed by the same anaesthesiologist skilled in the procedure. 

Thirty minutes after block application, patients were taken into the operating room with monitorization and we controlled periumbilical loss of sensation by pinprick test. 

Postoperative analgesia was planned as 1 g of paracetamol for 4 × 1 daily and tramadol 1 mg kg-1 as rescue therapy if visual analogue scale (VAS) score over 4 persisted after paracetamol dose.

Case 1: The defect size was 3 cm and surgery lasted 50 minutes. He just needed an extra dose of 100 µq fentanyl during the procedure. Patient and surgical satisfaction was good. Postoperative VAS scores were 1 and 3 (at first and sixth hour). He was discharged after 9 hours. 

Case 2: The defect size was 1 cm and surgery lasted 60 minutes. She needed an extra dose of 100 µq fentanil along with a total amount of 4 mg midazolam (other than the initial bolus dose). Patient satisfaction was moderate (she was anxious at the beginning of the surgery) and surgical satisfaction is good. Postoperative VAS scores were 3 and 4 (at first and sixth hour). She was discharged after 17 hours. 

Case 3: The defect size was discovered to be 7 cm and surgery lasted 80 minutes. We applied midazolam of 4 mg, fentanyl of 100 µg, and ketamine of 80 mg for extra sedation and analgesia throughout the surgery. Patient satisfaction was bad, and surgical satisfaction was moderate. Postoperative VAS scores were 6 and 4 (at first and sixth hour). He did not need rescue therapy other than paracetamol and he was discharged after 26 hours.

## Discussion

The sensorimotor innervation of the anterior abdominal wall is supplied by the ventral rami of the thoracolumbar spinal (T7-L1) segmental nerves. The thoracolumbar nerves course along the anterolateral wall eventually encroaching upon the lateral aspect of the rectus sheath. The nerves enter the lateral aspect of RAM and contribute to the formation of a nerve plexus that runs craniocaudal within the muscle and pierce the posterior border of RAM.^1^

Definite details regarding the course and distribution of the thoracolumbar nerves are lacking. For instance, a study by Courreges et al.^4^ showed that 30% of the population’s cutaneous branches of these nerves do not pierce the posterior wall instead they run anterior to RAM.^4^ Traditionally, RSB is performed via loss resistance technique. It could be the reason for its underutilization since RAM relates to vascular structures within the rectus sheath and visceral structures within the underlying peritoneal cavity making it particularly unsafe. However, with the use of ultrasonography, these concerns about local anaesthetic distribution, the accuracy of needle placement, especially concerning peritoneal puncture and visceral injury, can be achieved safely by real-time visualization. 

In practice, the time for the block application could be long. Because of the underlying peritoneal movement, it can be challenging to visualize all structures and the precise location of the needle tip. In our cases, the median duration of RSB application was 15 minutes. All blocks were performed by the same experienced anaesthesiologist.

The purpose of RSB is to anaesthetize the 9th, 10th, 11th intercostal nerves providing somatic anaesthesia to the anterior abdominal wall superficial to the peritoneum.^5^ For surgeries deep to the peritoneum, analgesia must be complemented with intravenous medication. This is why RSB can only be used as a sole anaesthetic technique for umbilical hernias with small size defects (below 4 cm as we hypothesize) without peritoneum involvement.^6^ Two of our cases had defect sizes less than 4 cm, one with 3 cm, and the other only 1 cm, and they both ended up with good surgeon and patient satisfaction. On the other hand, case 3 had a defect size of 7 cm and surgeon satisfaction was moderate. However, the patient was bad resulting in the need for more sedoanalgesia. Intraoperative discomfort of this case possibly affected VAS questionnaire as the first-hour VAS score was high although the sixth-hour VAS was 4.

The duration of surgery is also important. Although RSB appears to provide dense periumbilical analgesia, it has a short duration without catheter replacement. Webster et al.^7^ showed RSB to have denser postoperative analgesia compared to transversus abdominis plane block but with a shorter duration.^7^ In case 3, surgery extended more than expected. Therefore, dissociative anaesthesia was applied.

Besides, although the postoperative VAS score of the first hour was 6 in case 3, the VAS score of the 6th hour^4^ was the same with other cases. This result is well correlated with RSBs proven effect on postoperative analgesia regardless of defect size.^8^

Rectus sheath block with catheter placement has its place for postoperative analgesia relating to the anterior abdominal wall.^9^ But what about its use as a sole anaesthetic and why we should consider it in the first place with all the anatomical variations and shorter duration as regards general or neuraxial anaesthesia?

Rodriguez et al.^10^ presented a case of umbilical hernia with a patient having Wolff Parkinson White syndrome at which anaesthesia was managed solely by ultrasound-guided RSB. Since they should avoid sympathetic stimulation, regional anaesthesia was preferred over general anaesthesia. Furthermore, sympathetic blockage by neuraxial anaesthesia can lead to bradycardia and hypertension, and drugs for the treatment of these sıtuations could be detrimental in the scope of the patient's comorbidity. So what they needed was segmental anaesthesia with certain hemodynamic stability as they are well accomplished in this case by RSB.

Quek et al.^11^ compared a case of an infraumbilical hernia repair with ASA class 4, the patient had non-ischemic cardiomyopathy with an ejection fraction of 20%. They also used ultrasound-guided RSB successfully as a sole anaesthetic method.

Within the scope of high-risk patients, RSB can be an invaluable alternative with its segmental dense anaesthesia avoiding hemodynamic fluctuations commonly seen with general or neuraxial anaesthesia. Additionally, RSB can be used in the presence of relative coagulopathy and recent use of antiplatelets or anticoagulants. It would be precious for patients having coronary artery disease who should resume their therapy even perioperatively.

Rectus sheath block not only just eliminates the risks with high-risk patients but can also favor by allowing daily surgery since it preserves limb strength with no motor blockage thus allowing the patient to regain mobility and discharge early. This may also benefit regarding the patient's potential to exert deep vein thrombosis and pulmonary embolism and patients with respiratory compromise having a risk of atelectasis and pulmonary infection. Two of our patients with suitable defect sizes were discharged within 24 hours.

This is why it would be wise to improve the RSB technique as a valuable tool when there is no better.

All 3 of our patients did not have any significant obligation for RSB. We evaluated them for the effectiveness of RSB with the freedom to change protocols to qualify RSB without risk for the patients.

In the context of our cases, we manage to say sedoanalgesia is crucial with RSB.

The surgeons should be precise about the extent of the surgery. For instance, in our case 3, the surgical team extended through the peritoneum and we could only manage by dissociative anaesthesia. In a high-risk patient, it would be detrimental to change the anaesthetic plan intraoperatively without suitable preparation. Therefore, consulting and planning the case are essential for RSB. Since achieving a full cutaneous sensory block is also a challenge, local anaesthetic addition before incision can be recommended as an option.

## Conclusion

In a general view, ultrasound-guided RSB has the potential to be used as a sole anaesthetic for managing selected patients. We believe further studies are needed to protocolize for better technique and sedoanalgesia support. It could be wise to resurrect RSB from its overlooked literature.
